# Are Assistive Technologies Effective in the Management of Contractures in People with Stroke? - A Cochrane Review Summary with Commentary

**DOI:** 10.1177/10538135251323968

**Published:** 2025-05-03

**Authors:** Alex Todhunter-Brown

**Affiliations:** 1 Department of Nursing and Community Health, Glasgow Caledonian University, Glasgow, UK

**Keywords:** stroke, stroke rehabilitation, contracture, range of motion (articular), orthotic devices

## Abstract

**Background:**

Patients who have had a stroke are at risk of contractures, a shortening of muscles and other soft tissues. Muscle stretching, manually or delivered by other means, is a common strategy to manage contractures following stroke. A Cochrane Review by Mohammed Meeran et al. has investigated the effectiveness of assistive technology defined as a mechanical, electrical, or electromechanical device used to stretch or lengthen a muscle statically, dynamically, or cyclically for preventing or reducing contractures.

**Objective:**

To summarize the Cochrane Review by Mohammed Meeran et al. and comment on it from a rehabilitation perspective.

**Methods:**

The Cochrane Review authors identified randomised controlled studies (RCTs) which investigated the use of electrical, mechanical, or electromechanical devices to manage contractures in adults with stroke. RCTs had to compare these assistive technologies with no treatment, routine therapy, or another assistive technology. Passive range of motion (PROM) was the primary outcome.

**Results:**

Seven studies (294 participants) were included. Data from five studies (252 participants) comparing assistive technologies with routine care were meta-analyzed. The evidence was very uncertain about the effect of assistive technology on PROM, adverse events and other outcomes.

**Conclusions:**

There is insufficient evidence to support clinical decisions about the effectiveness of assistive technology in the management of contractures in people with stroke. Rehabilitation professionals should select strategies for management of contractures in patients with stroke based on an expert assessment of a patient's individual needs and preferences, and ensure regular re-assessment. High quality research is necessary in this field.

The aim of this commentary is to discuss from a rehabilitation perspective the Cochrane Review “Assistive technologies, including orthotic devices, for the management of contractures in adults after a stroke” by Mohammed Meeran et al. (2024)^1^, published on the Cochrane Library. This Cochrane Corner is produced in agreement with NeuroRehabilitation by Cochrane Rehabilitation with views* of the review summary author in the “implications for practice” section.

**Background:** Worldwide, stroke is the second leading cause of death and third most common cause of disability. Global data suggests there were 93.8 million stroke survivors in 2021 (Feigin et al., 2024). There is evidence of a substantial increase in stroke burden since 1990 and of greatest burden in low and low-middle income countries (LMIC) (Feigin et al., 2024). Around 80% of stroke survivors will be left with hemiplegia, which is a loss of motor function on one-side of the body, limiting movement and ability to carry out functional activities. The inability to move following stroke can result in contractures, which is a shortening of muscles and other soft tissues. Studies suggest that over half of stroke survivors may have some contracture (Mohammed Meeran et al., 2024). Contractures can potentially be prevented or reduced through stretching, though conclusive evidence of this in people with stroke is lacking (Gomez-Cuaresma et al., 2021).

**Assistive technologies, including orthotic devices, for the management of contractures in adults after a stroke**

(Mohammed Meeran et al., 2024)

**Objective:** The aim of this Cochrane Review was to investigate the effectiveness of “assistive technologies” in preventing or reducing contractures after stroke. Assistive technologies were defined as “a mechanical, electrical, or electromechanical device used to stretch or lengthen a muscle statically, dynamically, or cyclically”.

**What was studied and methods:** This was a Cochrane review of randomized controlled trials (RCTs). To be included in the review, RCTs had to compare assistive technologies (using the definition provided above) with no treatment, routine therapy or another assistive technology, in a population of adults with stroke. The primary outcome of interest was passive range of motion (PROM), at any joint, either measured using a goniometer or by taking a more indirect measurement of joint movement. Several secondary outcomes were assessed, including stiffness, pain, resting posture of limb, function, hygiene, carer burden, and adverse events. To be included, outcomes within a RCT had to have been assessed by someone blinded to the treatment group that participants were assigned to.

In May 2022 searches were conducted across a range of key databases (including MEDLNE, CINAHL, Embase, AMED, clinical trials registries, and the Cochrane Stroke Group specialized register of trials). The review was conducted using standard Cochrane methods, with risk of bias assessed using the Cochrane ROB1 tool and certainty of evidence judged using the GRADE approach.

**Results:** The review included seven studies (with 294 participants). All studies focused on the upper limb, with six targeting the wrist joint and the seventh the shoulder and elbow. Interventions included passive stretching, most commonly using splinting, and electrical stimulation, either alone or in combination with another assistive technology. Some assistive technologies were used for 30 min/day, whilst others were used for up to 12 h/day, with duration of intervention ranging from 4 to 12 weeks.

Data was only suitable for meta-analysis from five studies (252 participants), all of which compared assistive technology with routine care. The evidence was very uncertain about the effect of assistive technology on the primary outcome of PROM, with studies using a variety of measurements at different joints. The evidence was very uncertain about the effect of assistive technology on adverse events, with only two studies (53 participants) describing adverse events. There were little data available for secondary outcomes, with one study (81 participants) assessing stiffness, three studies (168 participants) assessing pain, and three studies (126 participants) using the Motor Assessment Scale; there was very low certainty in all findings. Key reasons for downgrading the certainty of evidence related to risk of bias, imprecision of the estimates and inconsistency of the results of individual studies.

**Conclusions**: The authors conclude that there is insufficient evidence to support clinical decisions about the effectiveness of assistive technology in the management of contractures in people with stroke. The authors identify a number of limitations within current research relating to the measurement of PROM and suggest that the dose / treatment intensity of interventions studies within RCTs has often been inadequate.

**Implications for practice in neurorehabilitation:** There is insufficient evidence to inform clinical decisions about the use of assistive technologies to manage contractures after stroke. Importantly, this does not mean that commonly used management strategies, such as use of splinting, are ineffective; but rather that the effectiveness is not known. People involved in the care of stroke survivors who have, or who are at risk of developing, contractures should select management strategies based on an expert assessment of a patient's individual needs and preferences. It is important that anyone involved in the assessment for, or use of, assistive technologies has appropriate skills and knowledge. Regular re-assessment, using appropriate, standardized approaches to measurement of range of movement of stroke survivors with, or at risk of, contractures is essential. The lack of high-quality research and knowledge relating to post-stroke contractures is notable, and rehabilitation professionals should seek opportunities to contribute to studies in this field.

## Notes

1. This summary is based on a Cochrane Review previously published in the Cochrane Database of Systematic Reviews 2024, Issue 9, Art. No.: CD010779, DOI: 10.1002/14651858.CD010779.pub2 (see www.cochranelibrary.com for information). Cochrane Reviews are regularly updated as new evidence emerges and in response to feedback, and Cochrane Database of Systematic Reviews should be consulted for the most recent version of the review.

* The views expressed in the summary with commentary are those of the Cochrane Corner author (different than the original Cochrane Review authors) and do not represent the Cochrane Library or Wiley.
